# Improving nutrition through biofortification: A review of evidence from HarvestPlus, 2003 through 2016

**DOI:** 10.1016/j.gfs.2017.01.009

**Published:** 2017-03

**Authors:** Howarth E. Bouis, Amy Saltzman

**Affiliations:** International Food Policy Research Institute, Washington, DC, United States

## Abstract

Biofortification is a feasible and cost-effective means of delivering micronutrients to populations that may have limited access to diverse diets and other micronutrient interventions. Since 2003, HarvestPlus and its partners have demonstrated that this agriculture-based method of addressing micronutrient deficiency through plant breeding works. More than 20 million people in farm households in developing countries are now growing and consuming biofortified crops. This review summarizes key evidence and discusses delivery experiences, as well as farmer and consumer adoption. Given the strength of the evidence, attention should now shift to an action-oriented agenda for scaling biofortification to improve nutrition globally. To reach one billion people by 2030, there are three key challenges: 1) mainstreaming biofortified traits into public plant breeding programs; 2) building consumer demand; and 3) integrating biofortification into public and private policies, programs, and investments. While many building blocks are in place, institutional leadership is needed to continue to drive towards this ambitious goal.

## Highlights

• Biofortification is proven to measurably improve human health and nutrition.
• Biofortified crops are now grown and consumed by more than 20 million people.
• Mainstreaming biofortified traits into plant breeding programs is a challenge.
• To reach scale, biofortification must be integrated in public and private programs.
• Institutional leadership is needed to reach 1 billion people by 2030.

**World Food Prize Winners 2016**

The World Food Prize is the foremost international award that honours outstanding individuals who have made vital contributions to improving the quality, quantity, or availability of food in the world (https://www.worldfoodprize.org/). This paper, authored by 2016 World Food Prize winner, Howarth Bouis and his colleague Amy Saltzman, both of the International Food Policy Research Institute, describes work under the HarvestPlus program on the global level on biofortification of staple foods to overcome micronutrient deficiencies. A companion paper in this issue by fellow 2016 winners, Jan Low, Maria Andrade, and Robert Mwanga of the International Potato Center describes work on biofortified orange-fleshed sweet potatoes in Africa.

## 1 Justification for biofortification

### 1.1 Introduction

Biofortification is a process of increasing the density of vitamins and minerals in a crop through plant breeding, transgenic techniques, or agronomic practices. Biofortified staple crops, when consumed regularly, will generate measureable improvements in human health and nutrition. This article extends the previously published theoretical framework for biofortification (Bouis et al., 2011b) and supporting evidence (Saltzman et al., 2013, Saltzman et al., 2015) to discuss delivery experiences and an action-oriented agenda for scaling biofortification to improve nutrition globally. Delivery experiences are discussed from the perspective of HarvestPlus, which leads a global interdisciplinary alliance of research institutions and implementing agencies in the biofortification effort.^1^ The evidence and building blocks for scale are in place; with sufficient institutional leadership, biofortification is poised to reach one billion people by 2030.

### 1.2 Comparative advantages

Micronutrient deficiencies afflict more than two billion individuals, or one in three people, globally (FAO et al., 2015). Such deficiencies occur when intake and absorption of vitamins and minerals are too low to sustain good health and development. Over the last 50 years, agricultural research for developing countries has increased production and availability of calorically dense staple crops, but the production of micronutrient-rich non-staples, such as vegetables, pulses and animal products, has not increased in equal measure. Non-staple food prices have increased steadily and substantially, making it more and difficult for the poor to afford dietary quality (Bouis et al., 2011a). In the long-term, increasing the production of micronutrient-rich foods and improving dietary diversity will substantially reduce micronutrient deficiencies. In the near term, consuming biofortified crops can help address micronutrient deficiencies by increasing the daily adequacy of micronutrient intakes among individuals throughout the lifecycle (Bouis et al., 2011b).

No single intervention will alleviate micronutrient deficiencies, and biofortification complements existing interventions, such as supplementation and industrial food fortification. Biofortification, however, has two key comparative advantages: its long-term cost-effectiveness and its ability to reach underserved, rural populations. Unlike the continual financial outlays required for supplementation and commercial fortification programs, an upfront investment in plant breeding yields micronutrient-rich biofortified planting material for farmers to grow at virtually zero marginal cost. Once developed, nutritionally improved crops can be evaluated and adapted to new environments and geographies, multiplying the benefits of the initial investment. Once the micronutrient trait has been mainstreamed into the core breeding objectives of national and international crop development programs, recurrent expenditures by agriculture research institutes for monitoring and maintenance are minimal.

Biofortified crops are also a feasible means of reaching rural populations who may have limited access to diverse diets or other micronutrient interventions. Target micronutrient levels for biofortified crops are set to meet the specific dietary needs of women and children, based on existing consumption patterns. Biofortification puts a solution in the hands of farmers, combining the micronutrient trait with other agronomic and consumption traits that farmers prefer. After fulfilling the household's food needs, surplus biofortified crops make their way into rural and urban retail outlets.

### 1.3 Cost-effectiveness

Ex-post cost-effectiveness data is currently available for orange sweet potato in Uganda, where biofortification was demonstrated to cost US$15–$20 per Disability Adjusted Life Year (DALY) saved, which the World Bank considers highly cost effective (World Bank, 1993, HarvestPlus, 2010).

Results of ex-ante cost-effectiveness studies have shown that for each of the country-crop-micronutrient combinations considered, biofortification is a cost-effective intervention based on cost per DALY saved, using World Bank standards (Meenakshi et al., 2010). Furthermore, the Copenhagen Consensus ranked interventions for reducing micronutrient deficiencies, including biofortification, among the highest value-for-money investments for economic development. For every dollar invested in biofortification, as much as US$17 of benefits may be gained (Hoddinott et al., 2012). The cost-effectiveness of any given intervention is dependent on the crop, micronutrient, and delivery country. The methodology for determining cost-effectiveness and specific case studies are discussed in greater depth elsewhere (Saltzman et al., 2013, Lividini and Fiedler, 2015, Asare-Marfo et al., 2013, de Brauw et al., 2015).

## 2 Nutritional bioavailability and efficacy evidence

Biofortified crops can improve human nutrition. To develop evidence of nutritional efficacy, nutritionists first measure retention of micronutrients in crops under typical processing, storage, and cooking practices to be sure that sufficient levels of vitamins and minerals will remain in foods that target populations typically eat (for summary results, see De Moura et al. (2015)). Genotypic differences in retention and concentrations of compounds that inhibit or enhance micronutrient bioavailability are considered. Nutritionists also study the degree to which nutrients bred into crops are absorbed, first by using models, then by direct study in humans in controlled experiments. Absorption is a prerequisite to demonstrating that biofortified crops can improve micronutrient status, but the change in status with long-term intake of biofortified foods must be measured directly. Therefore, randomized controlled efficacy trials are used to demonstrate the impact of biofortified crops on micronutrient status and functional indicators of micronutrient status (i.e. visual adaptation to darkness for vitamin A crops, physical activity and cognition tests for iron crops, etc.). Highlights are discussed below, and further detail is summarized in De Moura et al. (2014).

### 2.1 Iron crops

Iron nutrition research has demonstrated the efficacy of biofortified iron bean and iron pearl millet in improving the nutritional status of target populations. In Rwanda, iron-depleted university women showed a significant increase in hemoglobin and total body iron after consuming biofortified beans for 4.5 months (Haas et al., 2017). The efficacy of iron pearl millet was evaluated in secondary school children from Maharashtra, India. A significant improvement in serum ferritin and total body iron was observed in iron-deficient adolescent boys and girls after consuming biofortified pearl millet flat bread twice daily for four months. The prevalence of iron deficiency was reduced significantly in the high-iron biofortified pearl millet group. Those children who were iron deficient at baseline were significantly (64%) more likely to resolve their deficiency by six months (Finkelstein et al., 2015).

### 2.2 Vitamin A crops

Vitamin A bioavailability studies found efficient conversions from provitamin A to retinol, the form of vitamin A used by the body. Efficacy studies demonstrated that increasing provitamin A intake through consuming vitamin A-biofortified crops results in increased circulating beta-carotene, and has a moderate effect on vitamin A status, as measured by serum retinol. Consumption of orange sweet potato (OSP) can result in a significant increase in vitamin A body stores across age groups (Haskell et al., 2004; Low et al., 2007; van Jaarsveld et al., 2005).

The primary evidence for the effectiveness of biofortification comes from OSP, assessed through a randomized controlled trial. The OSP intervention reached 24,000 households in Uganda and Mozambique from 2006 to 2009 with adoption rates of OSP greater than 60% above control communities (Hotz et al., 2012a, Hotz et al., 2012b). Introduction of OSP in rural Uganda resulted in increased vitamin A intakes among children and women, and improved vitamin A status among children – a decrease in the prevalence of low serum retinol by 9 percentage points. Women who got more vitamin A from OSP also had a lower likelihood of having marginal vitamin A deficiency (Hotz et al., 2012a). Recent research on the health benefits of biofortified OSP in Mozambique showed that biofortification can improve child health; consumption of biofortified orange sweet potato reduced the prevalence and duration of diarrhea in children under five (Jones and de Brauw, 2015). For additional information on the development and delivery of OSP, see Low et al. (2017, this issue).

Biofortified provitamin A maize is an efficacious source of vitamin A when consumed as a staple crop. An efficacy study conducted in Zambia with 5–7-year-old children showed that, after three months of consumption, the total body stores of vitamin A in the children who were in the orange maize group increased significantly compared with those in the control group (Gannon et al., 2014). Consumption of orange maize has been demonstrated to improve total body vitamin A stores as effectively as supplementation (Gannon et al., 2014), and significantly improve visual function in marginally vitamin A deficient children (Palmer et al., 2016).

To date, only a small provitamin A cassava efficacy study has been completed in Eastern Kenya with 5–13-year-old children. This trial demonstrated small but significant improvements in vitamin A status, measured both by serum retinol and *beta*-carotene, in the yellow cassava versus the control group (Talsma et al., 2016). A larger-scale efficacy trial is underway in Nigeria.

### 2.3 Zinc crops

Zinc studies have demonstrated that zinc in biofortified wheat is bioavailable (Rosado et al., 2009). Because plasma zinc concentration, the biomarker widely used to estimate zinc status, has limitations in measuring changes in dietary zinc, foundational research to identify and test more sensitive biomarkers is underway. These biomarkers will be tested in the zinc rice and wheat efficacy trial scheduled for 2017. A recent study showed that DNA strand breaks are a sensitive indicator of modest increases in zinc intake, such as the amount of additional zinc that might be delivered by a biofortified crop (King et al., 2016).

### 2.4 Future areas of investigation

Areas for further research include robust new trials that test the efficacy of biofortified crops for a wider range of age and gender groups, including infants, and over a longer time period (for example, prior to conception through infancy). Other research will test the efficacy of consuming several different biofortified crops, each providing different vitamins and/or minerals to the food basket. Nutritionists agree that biofortified crops can improve nutritional status in micronutrient-deficient populations, but additional research is needed, using other, more sensitive biochemical indicators, as well as functional indicators, to more fully understand the health impact of consuming biofortified foods.

## 3 Crop development

Plant breeding can increase nutrient levels in staple crops to target levels required for improving human nutrition, without compromising yield or farmer-preferred agronomic traits. The crop development process entails screening germsplasm for available genetic diversity, prebreeding parental genotypes, developing and testing micronutrient-dense germplasm, conducting genetic studies, and developing molecular markers to lower the costs and quicken the pace of breeding. After promising lines have been developed, they are tested in several locations across target environments to determine the genotype x environment interaction (GxE) – the influence of the growing environment on micronutrient expression. Robust regional testing enables reduced time-to-market for biofortified varieties.

**Table 1:** Breeding targets (parts per million).

**Provitamin A**		**Sweet potato**	**Maize**	**Cassava**
	Baseline micronutrient content	2	0	0
	Additional content required	30	15	15
	**Final target content**	**32**	**15**	**15**
**Iron**		**Beans**	**Pearl Millet**	
	Baseline micronutrient content	50	47	
	Additional content required	44	30	
	**Final target content**	**94**	**77**	
**Zinc**		**Rice**	**Wheat**	
	Baseline micronutrient content	16	25	
	Additional content required	12	12	
	**Final target content**	**28**	**37**	

### 3.1 A conceptual framework for breeding biofortified germplasm

**Fig. 1:**
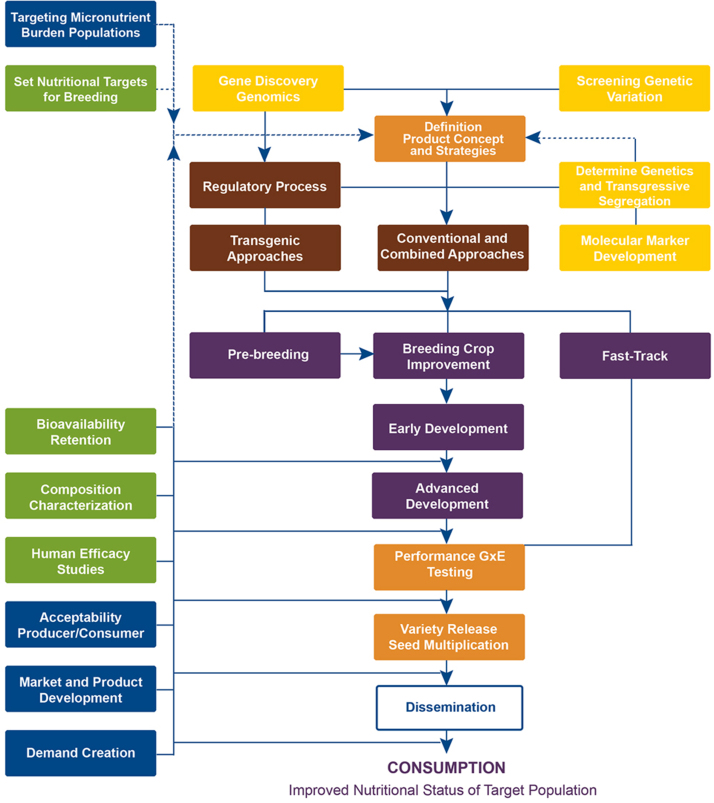
Crop development framework. Source: HarvestPlus.

Crop improvement activities for biofortification focus, first, on exploring the available genetic diversity for iron, zinc, and provitamin A carotenoids (yellow boxes). At the same time or during subsequent screening, agronomic and end-use features are characterized. The objectives when exploring the available genetic diversity are to identify: (1) parental genotypes that can be used in crosses, genetic studies, molecular-marker development, and parent-building, and (2) existing varieties, pre-varieties in the release pipeline, or finished germplasm products for “fast-tracking.” Fast-tracking refers to releasing, commercializing, or introducing genotypes that combine the target micronutrient density with the required agronomic and end-use traits so they can be delivered without delay.

If variation is present in the strategic gene pool (only in unadapted sources), pre-breeding is necessary prior to using the trait in final product development; if variation is present in the adapted gene pool, the materials can be used directly to develop competitive varieties (purple boxes). Most breeding programs simultaneously conduct pre-breeding and product enhancement activities to develop germplasm combining high levels of one or more micronutrients.

The next breeding steps involve developing and testing micronutrient-dense germplasm, conducting genetic studies, and developing molecular markers to facilitate breeding. Genotype x environment interaction (GxE) – the influence of the growing environment on micronutrient expression – is then determined at experiment stations and in farmers’ fields in the target countries (orange boxes). The most promising varieties are selected for multi-locational testing over multiple seasons by national research partners, and then are submitted to national government agencies for testing for agronomic performance and release, a process which typically takes two years, sometimes more.

### 3.2 Transgenic approaches

In crops where the target nutrient does not naturally exist at the required levels in the tens of thousands of varieties in germplasm banks, transgenic plant breeding is a promising approach to produce biofortified crops with the desired nutrient and agronomic traits. For example, transgenic iron and zinc rice has been developed and tested in confined field trials that can provide 30% of the EAR for both nutrients (Trijatmiko et al., 2016). Golden rice, which contains beta carotene, can provide more than 50% of the EAR for vitamin A. Despite being available as a prototype since early 2000, however, Golden Rice has not been introduced in any country, in large part due to highly risk-averse regulatory approval processes (Wesseler and Zilberman, 2014). While these transgenic varieties have tremendous nutritional potential, release to farmers is several years in the future, and depends on approval through national biosafety and regulatory processes.

Conventional breeding, rather than transgenic breeding, is used in all of the crops released or in the near pipeline for HarvestPlus programs. Because conventional breeding does not face the same regulatory hurdles and is widely accepted, HarvestPlus considers it to be the fastest route to getting more nutritious crops into the hands of farmers and consumers. This article focuses on the evidence developed for conventionally-bred biofortified crops.

### 3.3 International nurseries/global testing

HarvestPlus has used two strategies to shorten time to market for biofortified crops: 1) identifying ***adapted*** varieties with significant micronutrient content for release and/or dissemination as “fast track” varieties, while varieties with target micronutrient content are still under development, and 2) deploying multi-location Regional Trials across a wide range of countries and sites to accelerate release processes by increasing available performance data of elite breeding materials. Regional Trials also include already-released biofortified varieties and generate data on their regional performance, in order to take advantage of regional variety release systems such as under SADC (Southern African Development Community). Such regional agreements harmonize seed regulations of member countries and allow any variety that is tested, approved, and released in one member country to be released simultaneously in other member countries with similar agro-ecologies.

### 3.4 Low-cost, high throughput methods

Biofortification breeding required developing or adapting cost-effective and rapid high throughout analytical techniques for micronutrients, as thousands of samples need to be tested for mineral or vitamin content each season. These trait diagnostics include near-infrared spectroscopy (NIRS) and colorimetric methods for carotenoid analysis. For mineral analysis, X-ray fluorescence spectroscopy (XRF) emerged as the method of choice, as it requires minimal pre-analysis preparation and allows for non-destructive analysis (Paltridge et al., 2012a, Paltridge et al., 2012b).

### 3.5 Releases of biofortified crops

**Fig. 2:**
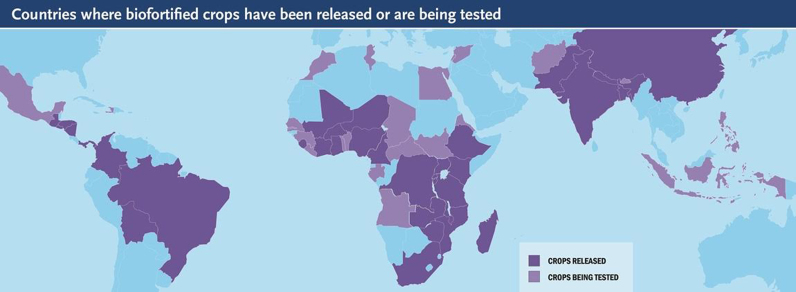
Biofortified crop map (Jan 2017). Source: HarvestPlus.

## 4 Delivery experiences in target countries

**Table 2:** Number of households reached in target countries annually, 2012–2015 (x1000).

**Country-Crop**	**2012**	**2013**	**2014**	**2015**
Iron Bean Rwanda	105	609	332	453
Iron Bean DR Congo	60	241	128	175
Iron Bean Uganda	29	69	43	39
Iron Pearl Millet India	30	40	62	125
Provitamin A Maize Zambia	0	11	104	126
Provitamin A Cassava Nigeria	0	106	360	528
Provitamin A Cassava DR Congo	0	25	75	127
Provitamin A Orange Sweet Potato Uganda	33	76	107	132
Zinc Wheat India	0	1	6	36
Zinc Wheat Pakistan	0	0	0	3
Zinc Rice Bangladesh	0	1	18	160
**Total**	**257**	**1,179**	**1,235**	**1,865**

Target countries represent a variety of market environments for biofortified crops, from a primarily commercial private sector approach (India, Zambia), to various mixed public-private delivery systems (Bangladesh, Nigeria, Rwanda, Uganda), to primarily public or informal market systems (DR Congo). Progress in the integration of biofortified crops into the seed and food value chains in target countries is discussed below, using case studies to discuss how HarvestPlus and its partners have strengthened seed systems, created knowledge and demand, and expanded partnerships to ensure the future sustainability of biofortification. Examples of delivery experiences are presented for vegetatively propagated, self-pollinated, and hybrid crops. Impact and effectiveness data are presented where available; for countries where these studies are still underway, evidence is primarily based on information captured through monitoring systems.

### 4.1 Vegetatively propagated crops

Vegetatively propagated crops – those for which farmers plant stems, tubers or vines rather than seeds – typically have seed systems characterized by small, informal (rather than commercial) actors. Planting materials are perishable, expensive and bulky to transport over long distances, and must be replanted within several days of harvesting. The lack of commercial private sector participation creates both a challenge and an opportunity for producing planting materials of biofortified crops like orange sweet potato (distributed as vines) and provitamin A yellow cassava (distributed as stem cuttings). See Low et al. (2017, this issue) for additional evidence from OSP delivery.

#### 4.1.1 Cassava in Nigeria and DR Congo

In parallel with strengthening the seed system through both community-based and commercial stem production, awareness of and demand for biofortified crops must be created simultaneously. In the case of provitamin A yellow cassava, extension to farmers was at the forefront of this effort. Initially, free bundles of stems were distributed to farmers, and accompanied by agronomic training and nutrition information. In the following season, farmers who received free stems were required to distribute an equal amount of free stems to two additional farmers, dramatically lowering delivery costs. This promotional strategy was effective in reaching vulnerable populations who typically do not have market access to improved varieties for planting. It also piqued interest and allowed farmers in a low-risk way to test a new product. Many of the farmers who received and planted free stems liked the yellow cassava and are now buying additional stems from commercial traders.

In 2015, HarvestPlus estimated that about 75% of all biofortified harvested roots were consumed on farm, as many households were not yet producing excess from the stem packs they received for trial. Increased commercialization is expected going forward. As farmers began to produce yellow cassava in excess of their household food security needs, HarvestPlus and its partners have worked to increase awareness and demand from the food market for biofortified cassava. These efforts include consumer marketing via print, radio, and television media (even feature-length movies), and market development efforts by linking commercial food processing investors to supplies of yellow cassava roots.

### 4.2 Self-pollinated crops

Self-pollinated crops – those which produce seed true to their parent characteristics – can be replanted year after year. While farmers do need to periodically replace their seed to maintain its desirable agronomic traits, the possibility of self-production for seed typically limits private sector investment in producing seed for self-pollinated crops.^4^ In many countries, the public sector instead multiplies and distributes self-pollinated seed, and further farmer-to-farmer dissemination is common. Self-pollinated biofortified crops include iron beans, delivered in Rwanda and Democratic Republic of Congo, zinc rice in Bangladesh, and zinc wheat in India and Pakistan.

Delivery has progressed most quickly in Rwanda, where initial public sector investments have now spurred private sector interest in meeting growing demand for iron bean seed. Significant delivery has also taken place in Bangladesh, where demand is driven by the zinc rice varieties that have attractive agronomic traits, including a short duration variety that allows for production of a third crop between the wet and dry season rice crops. Delivery of zinc wheat in India and Pakistan is just beginning. In India, zinc wheat is predominantly marketed by the private sector as truthfully labeled seed (TLS), and six private seed companies had incorporated zinc wheat into their product lines. In Pakistan, the first zinc wheat variety was released in 2016, and delivery through public and private sector partners is now underway.

#### 4.2.1 Beans in Rwanda and DR Congo

In Rwanda, HarvestPlus worked closely with the Rwanda Agriculture Board (RAB) to facilitate production of bean seed through contracted farmers, cooperatives, and small seed companies. From 2011 to 2015, HarvestPlus procured about 80% of its certified seed through registered seed farmers under the supervision and certification of RAB, with the remainder being produced through contracts with local seed companies. To increase available seed for the 2015 planting season and beyond, HarvestPlus partnered with established local and regional seed companies for seed multiplication, with RAB certifying the biofortified seed. HarvestPlus and its partners also proposed a new seed class, “Declared Quality Seed” (DQS) or Certified II seed, first in Rwanda and then in DRC. DQS is produced from certified seed and is priced between certified seed and grain, bridging a price gap for farmers who are inclined to plant recycled grain rather than purchase certified seed.

Farmers initially accessed iron bean seed either in small quantities through direct marketing (via established agrodealers or in local markets) or in larger quantities through a payback system that also included cooperatives. By the end of 2014, marketing data showed that an increasing number of farmers were purchasing seed, a trend that is expected to continue. Farmer-to-farmer dissemination is also an important delivery channel; an impact assessment conducted in 2015 found that nearly half of farmers growing iron bean had received their planting material from a person in their social network (Asare-Marfo et al., 2016).

Because the iron trait is invisible and iron beans are not easily distinguished from conventional varieties, the primary approach has been to gain market share for biofortified beans due to their superior agronomic and consumption qualities. Over time, a high percentage of the total national supply of beans is expected to contain the biofortified trait, allowing access to additional iron for much of the population. HarvestPlus and its partners have used a variety of delivery methods, including “swapping” biofortified seed for conventional seed, to ensure a high rate of farmer trial and adoption. Only five years after the first iron bean release, iron beans make up more than 10% of national bean production in Rwanda (Asare-Marfo et al., 2016).

#### 4.2.2 Rice in Bangladesh

At the core of the Bangladesh strategy are rice varieties with attractive agronomic properties and a robust farmer demonstration program. One released zinc rice for the wet season (BRRI dhan 64) is a short duration variety (100 days as compared with 140 days), which allows production of a third crop of lentils or other food between wet and dry season rice crops. Other biofortified zinc rice varieties carry different farmer-preferred agronomic traits, like high height at maturity, which is beneficial for flooded areas in Southern Bangladesh. A robust demonstration program provides farmers a chance to observe these new varieties, as well as training on growing the biofortified rice and the health benefits of zinc.

Seed is produced by both the private and the public sector. A private seed association called SeedNet produces truthfully labeled seed alongside the foundation and certified seed produced by government entities. HarvestPlus initially both guarantees a market for a portion of the private sector production and subsidizes the price for any seed that the private sector markets directly to consumers. Free seed is distributed by NGO and government partners in small seed packs, and all free seed recipients agree to pass on the same amount of seed to three neighboring farmers in the subsequent season. As an increasing amount of zinc rice is available on the market, efforts to increase consumer and miller awareness have increased, including outreach via SMS and programs on local television and community radio channels.

### 4.3 Hybrid crops

Hybrid crops – those for which seed must be replaced each year to maintain the same yield and agronomic traits – offer the most potential for private sector commercialization. While utilizing the private sector for delivery may lead to long-term sustainability, the speed of private sector uptake is dependent on their assessment of demand. Therefore, the activities of biofortification proponents must focus on targeted demand creation for both farmers and consumers.

#### 4.3.1 Maize in Zambia

Because private seed companies dominate the hybrid maize seed market in Zambia, upon release, biofortified hybrid varieties were licensed to companies for commercialization of seed production and distribution. As biofortified maize is scaled up to reach more households in more provinces, the main challenge is to ensure extensive distribution through private networks to outlying areas. Because many rural households purchasing from agrodealers cannot afford to buy large quantities of seed, HarvestPlus is working with the private seed companies to ensure that large quantities of smaller, affordable pack sizes will be available. HarvestPlus also partners with the Zambia National Farmers Union and government extension services to disseminate information to farmers about the availability of vitamin A maize seed in their local areas. The inclusion of orange maize seed in the Zambian government's Farmer Input Support Program (FISP) has further facilitated access to orange maize, including for vulnerable households. FISP provides at least a 50% subsidy for maize seed and fertilizer to farmers considered economically disadvantaged. The quantity of orange maize seed distributed under FISP grew by 400% between the first and second year of inclusion in the program.

A central element of the delivery strategy is to create awareness and acceptance of orange maize through the use of social marketing campaigns and advertisements placed in public media, including TV, radio, newspapers, and popular music. Educational and awareness-creation activities stimulate consumer demand for orange maize products, while engagement with the private sector helps meet growing consumer demand.

To further stimulate cultivation of orange maize, creating markets for surplus production was essential, considering that 20–50% of rural households sell maize after satisfying their own food needs. HarvestPlus therefore links major grain buyers to farmers and offers grain samples to millers and food processors interested in incorporating orange maize in their product lines. The multi-lateral AgResults initiative also incentivizes millers to produce and market vitamin A maize products. Strong interest from farmers and food processors encourages increased private sector seed production.

#### 4.3.2 Pearl millet in India

Crop development and delivery in India is implemented through public and private sector partnerships. In crop development, the International Crops Research Institute for the Semi-Arid Tropics (ICRISAT) supplies parental materials/breeder seeds for next stage seed multiplication. Partners now testing and developing their iron pearl millet varieties for seed sales include 15 private seed companies, 2 public seed companies and 5 public organizations. HarvestPlus supports ICRISAT to develop high iron hybrid parental lines and to test hybrids with farmer-preferred traits, including of course high yields. This unique crop development arrangement supports and encourages companies to develop their own biofortified varieties for their target market segments. This approach is expected to more quickly increase the number and range of biofortified varieties available in the years to come.

#### 4.3.3 Lessons learned from delivery

While delivery experiences vary widely by country and seed system, a few common themes have emerged from the delivery experience. First, multiplication of sufficient planting material is a crucial first step – without planting material to “prime the pump”, there are no biofortified crops. HarvestPlus and its partners have focused on both strengthening capacity in the public and private sector to produce high quality seed and reducing risk, to ensure that quality planting material is available for farmers. Second, demonstration trials have been key demand drivers at the farm level. Decentralized field demonstrations and the availability of small promotional seed packs have allowed interested farmers to view and try the new product without taking on a great deal of risk in cultivating a crop for which the market has not yet been tested. Third, nutrition messaging aimed at both men and women has also been key, and in general, involving women farmers has led to increasing demand for biofortified crops. While many biofortified crops are acceptable to farmers and consumers without further information about their nutrition traits, nutrition information helps ensure that the biofortified foods are integrated into child diets (Birol et al., 2015). Finally, multi-stakeholder platforms are crucial to scaling up the early uptake and success of biofortified crops. In target countries, there has been rapid acceptance of biofortification by government entities, and national governments have proactively integrated it into their agriculture and nutrition policies. Integrating private and public sector actors and interests around shared goals reduces barriers to scaling.

## 5 Building blocks for global delivery

For biofortification to reach scale and be truly sustainable, a number of institutions must become involved in establishing an enabling environment. This includes recognition of biofortification among global normative and regulatory agencies, integration into development policies and programs funded by multi-lateral institutions, uptake by private sector entities, and incorporation into development programs being implemented on the ground, both in target countries and beyond. This enabling environment is essential to encourage the scaling up of biofortified crops and to support national-level actors in various spheres.

### 5.1 Standards and regulatory

Efforts are underway to integrate biofortification into global standards and guidelines, such as the Codex Alimentarius, the food standards-setting agency administered jointly by the World Health Organization (WHO) and the Food and Agriculture Organization of the United Nations (FAO) and recognized by the Sanitary and Phytosanitary Agreement (SPS) of the World Trade Organization (WTO) as its reference organization. Progress toward the development of a definition and standards for biofortification within the Codex Alimentarius continues. Once adopted, the internationally-recognized Codex reference standard helps to facilitate cross-border marketing of biofortified crops and food, to standardize labeling and health claims, and to reduce the incidence of false claims.

### 5.2 Multi-lateral institutions

Beyond their individual investments and activities, multi-lateral institutions, including the World Bank, the African Development Bank, the World Food Programme, and the World Health Organization, collectively influence national government policymakers and operational partners.

The World Bank is now implementing a number of projects supporting biofortification, including the Multisectoral Food Security and Nutrition Project in Uganda, which is accelerating the scale-up of orange sweet potato and iron beans. As a convener of development partners, the Bank plays an important role in encouraging nutrition-sensitive agricultural approaches, including biofortification, in arenas such as the Global Donor Platform for Rural Development. The African Development Bank's new “Banking on Nutrition” technical partnership is implementing a multi-sectoral and integrated approach to nutrition interventions, including the integration of biofortified crops. The World Food Programme's (WFP) Purchase for Progress program is very interested in local purchase of biofortified crops, and partnerships are being developed in several countries. For example, in Rwanda, local iron bean production is purchased and stored in WFP warehouses for later emergencies.

In 2017 the WHO Nutrition Guidance Expert Advisory Group is expected to issue a recommendation and guidelines on biofortification as a public health nutrition intervention. One step in the process will be the publication of papers discussed in 2016 at an expert consultation held at the New York Academy of Sciences.

### 5.3 Private sector

As crop development programs increase the number of released varieties of biofortified crops, seeds from these varieties must be made available to farmers. In countries with robust private seed systems that reach smallholder farmers, private seed companies are a natural partner. In some cases, HarvestPlus has brokered agreements between seed companies and interested NGOs or government entities to ensure that there will be a market for the seed produced by the private sector, reducing the risk associated with that private sector investment. While the private sector has predominantly taken up hybrid crops, interest in a wider variety of crops has increased as the business case has been developed. Involving private sector seed companies not only in marketing, but also in developing and testing biofortified varieties, shortens the time to market and lays the groundwork for sustainability.

Food processing companies play an important role in developing the food product value chain for biofortified crops. Small and medium-size companies can play a role in creating demand for biofortified grain and food even before supplies reach scale. For some crops and countries, like Nigeria cassava, the food value chain is dominated by small and medium-size food processors. While the interest of multi-national companies is slower to develop, several are now testing biofortified crops in their food products. These companies also contribute to the evidence base on vitamin and mineral retention by assessing different processing methods for biofortified crops.

### 5.4 NGOs

While private sector participation is essential in creating sustainable markets for biofortified seed and foods, NGOs remain important in delivering this nutrition intervention to vulnerable households. The existing global partnership between World Vision and HarvestPlus is an example of how a leading development NGO can incorporate biofortified crops into its existing agricultural programs, linking them to health and nutrition programs. While HarvestPlus provides technical assistance, World Vision takes the lead in delivery, with activities now in 15 countries. This type of partnership, whereby biofortified crops are integrated into existing agriculture and nutrition projects or included in collaboratively developed new projects, will continue to be important to reach the most vulnerable households, which may also be the most likely to suffer from micronutrient deficiencies.

### 5.5 Moving beyond target countries to partnership country strategies

Outside of target countries, HarvestPlus has invested in, advocated for, and now works closely with government-sponsored biofortification programs in Brazil, China, and India. Through the HarvestPlus Latin American and Caribbean (LAC) program, led by the Brazilian Ministry of Agriculture's Research Corporation (EMBRAPA), HarvestPlus provides technical assistance and support to government-driven biofortification programs in Bolivia, Colombia, Guatemala, Haiti, Nicaragua, and Panama and is exploring efforts in several additional countries. As biofortification gains momentum, this type of partnership approach is essential. While HarvestPlus will continue to provide technical assistance and promote linkages between organizations, other organizations and actors will increasingly take the lead in delivery on the ground.

## 6 A future vision: institutional leadership to drive and guide mainstreaming

To reach its full potential, biofortification must be integrated as a core activity within a range of global institutions. This will require three critical elements.

1. *Supply*: Agricultural research entities, both public and private, come to recognize high mineral and vitamin content as core plant breeding objectives; varietal release committees make minimum levels of minerals and vitamins a requirement for approval for release (in addition to the standard agronomic traits, such as high yield).
2. *Policy*: A wide range of national and international public officials come to recognize the significant impact of biofortification for improving and sustaining public health, as well as the high economic return to investments in biofortification and the legitimacy conferred by international recognition (especially by standards bodies).
3. *Demand*: Both rural and urban consumers come to see the value of, and demand, high mineral and vitamin content in their staple foods.

### 6.1. Supply

The key to continued supply of biofortified crops is to move beyond a biofortification-focused breeding program, with funding specifically for biofortified crops, to mainstream the nutrient traits into all relevant crop pipelines and the best crop backgrounds being developed by CGIAR centers and National Agricultural Research Systems (NARS). Recent progress in developing molecular markers will help facilitate mainstreaming (Babu et al., 2013, Swamy et al., 2016). As new varieties are developed and released, they should include the biofortified trait as a matter of standard practice.

### 6.2. Policy

Significant progress has already been made in integrating biofortification into regional and national policies. At the Second International Conference on Nutrition (ICN2) held in Rome in 2014, high-level government representatives from Bangladesh, Malawi, Nigeria, Pakistan, and Uganda highlighted the role of biofortification in their national strategies to end malnutrition by 2025. More than 20 additional countries, including Colombia, Panama, Rwanda, and Zambia, have included biofortified crops in their national agriculture and nutrition plans. Regional and global processes, like the African Union's Comprehensive Africa Agriculture Development Program (CAADP) and the Scaling Up Nutrition (SUN) Movement are building an enabling environment for biofortification. Governments are so positive about the impacts of the lead biofortified varieties introduced into their countries that they have requested additional biofortified crops be introduced. These efforts must continue to integrate biofortification in policies at all scales.

### 6.3. Demand

The potential benefit of increasing market demand for biofortified crops – and thereby making them more attractive to farmers to grow – must be balanced with the aim of biofortified foods reaching populations suffering from micronutrient deficiency. To ensure that biofortfied crops are sustainable, however, both rural and urban consumers must demand high mineral and vitamin content in their staple foods. As discussed in the delivery section, superior agronomic traits and nutrition messaging drive demand from rural smallholders.

### 6.4 . Conclusion

Scaling will require building new and expanding existing partnerships, maintaining engagement, and increasing partner capacity. More than 100 HarvestPlus delivery partners have trained thousands of extension staff on agronomic practices and nutrition messages for biofortification, and developed technical packages for partners to use in delivery programming. Going forward, HarvestPlus will add new and diverse partners, including food processing companies and retailers, UN agencies, regional organizations, and innovative financing mechanisms and development banks.

To reach one billion people by 2030, however, biofortification must move beyond HarvestPlus. Policymakers must give higher priority to the role of agriculture to improve health. National governments and multilateral institutions must ensure that biofortification is included on the nutrition agenda. Public and private sector breeding partners must mainstream the biofortified trait across their product lines. Food processors and other actors along the value chain must include biofortified crops in their products. Only through a collaborative effort that reaches across the value chain will biofortification become business as usual, and the vision of reaching one billion become a reality.

## Footnotes

1 HarvestPlus is one component of the CGIAR Research Program on Agriculture for Nutrition and Health (A4NH). The CGIAR is a global agriculture research partnership for a food secure future. Its science is carried out by its 15 research centers in collaboration with hundreds of partner organizations. The HarvestPlus program is administered under a joint venture agreement by two of these centers, the International Center for Tropical Agriculture (CIAT) and the International Food Policy Research Institute (IFPRI). The primary role of HarvestPlus is to catalyze, coordinate, and conduct oversight over a complex set of interdisciplinary activities by a large number of partner institutions, that lead to reductions in mineral and vitamin deficiencies through biofortification. HarvestPlus has invested more than $300 million in biofortification activities during 2003–2016. Principal investors in HarvestPlus currently include the UK Government, Bill & Melinda Gates Foundation, the US Government's Feed the Future initiative, the EU Commission, and donors to A4NH.

4 For crops with a low seed rate, like pearl millet, farmers are more likely to purchase seed annually. An open-pollinated variety of biofortified iron pearl millet, which combines the iron trait with 10% higher yield, has been successfully deployed through the private sector in India, where farmers generally purchase seed annually.
